# Trends in birth attendants in Sudan using three consecutive household surveys (from 2006 to 2014)

**DOI:** 10.3389/fgwh.2023.1012676

**Published:** 2023-08-29

**Authors:** Noon Altijani, Mustafa Khogali, Lisa Hinton, Charles Opondo, Eman Eljack, Marian Knight, Manisha Nair

**Affiliations:** ^1^ Nuffield Department of Population Health, University of Oxford, Oxford, United Kingdom; ^2^ School of Medicine, Ahfad University for Women, Omdurman, Sudan; ^3^ The Healthcare Improvement Studies Institute, University of Cambridge, Cambridge, United Kingdom; ^4^ National Perinatal Epidemiology Unit, Nuffield Department of Population Health, University of Oxford, Oxford, United Kingdom; ^5^ Health Systems Strengthening and Malaria Program Management Unit, Federal Ministry of Health, Khartoum, Sudan

**Keywords:** trends, skilled health personnel, skilled birth attendant, Sudan, household surveys, maternal mortality

## Abstract

**Introduction:**

Improving maternal health and survival remains a public health priority for Sudan. Significant investments were made to expand access to maternal health services, such as through the training and deployment of providers with varying skills and competencies to work across the country. This study investigates trends in the coverage of different birth attendants and their relationship with the maternal mortality ratio (MMR).

**Methods:**

Trend analyses were conducted using data from the 2006, 2010, and 2014 Sudan Household surveys. Three categories of birth attendants were identified: (1) skilled birth attendants (SBA) such as doctors, nurse-midwives, and health visitors, (2) locally certified midwives, and (3) traditional birth attendants (TBA). Multivariable logistic regression models were used to examine trends in SBAs (vs. locally certified midwives and TBAs), locally certified midwives (vs SBAs and TBAs), and SBAs and locally certified midwives by place of birth (health facility and home). The analyses were adjusted for potential confounders. An ecological analysis was conducted to assess the relationship between birth attendants by place of birth and MMR at the state level.

**Results:**

Births by 15,848 women were analysed. Locally certified midwives attended most births in each survey year, with their contribution increasing from 36.3% in 2006 to 55.5% in 2014. The contributions of SBAs and TBAs decreased over the same period. In 2014 compared with 2006, births were more likely to be attended by a locally certified midwife (aOR: 2.19; 95%CI: 1.82–2.63) but less likely to be attended by a SBA (aOR: 0.46; 95%CI: 0.37–0.56). The decrease in SBA was more substantial for births taking place at home (aOR: 0.17; 95%CI: 0.12–0.23) than for health facility births (aOR: 0.45; 95%CI: 0.31–0.65). In the ecological analysis 2014–2016, the proportion of births attended by SBA in health facilities correlated negatively with MMR at state level (rho −0.55; *p*: 0.02).

**Conclusion:**

This analysis suggests that although an improved coverage of maternal health with locally certified midwives has been observed, it has not provided the skill level reached by SBA. SBAs working in facility settings were a key correlating factor to reduced maternal mortality. Urgent action is needed to improve access to SBAs in health facilities, thereby accelerating progress in reducing maternal mortality.

## 1. Introduction

Many low- and middle-income countries continue to report high maternal mortality ratios (1). This is despite national and global efforts to meet targets set out in the sustainable development goals (SDG) and the strategies for ending preventable maternal mortality (EPMM) (2–4). Target 3.1 of the SDGs, and the primary target of EPMM both aim to reduce the global maternal mortality ratio (MMR) to less than 70 per 100,000 live births by 2030. The EPMM also has a supplementary, country-specific target that is for no country to have an MMR greater than 140 per 100,000 live births by 2030. For pregnant women, the risk of maternal death is greatest around labour, birth, and the immediate postpartum period given the potential for complications (5). Thus, a key strategy to reduce maternal deaths has been to ensure the presence of a skilled health personnel, also known as a skilled birth attendant (6) around the time of birth to safeguard physiological birth and/ or manage complications (3, 7–10). These skilled health personnel are defined as “competent maternal and newborn health (MNH) professionals educated, trained and regulated to national and international standards. They are competent to: (i) provide and promote evidence-based, human-rights-based, quality, socioculturally sensitive and dignified care to women and newborns; (ii) facilitate physiological processes during labour and delivery to ensure a clean and positive childbirth experience; and (iii) identify and manage or refer women and/or newborns with complications.” (11).

The proportion of skilled health personnel attending childbirth, along with the maternal mortality ratio (MMR) are used to monitor countries’ progress towards target 3.1 of the SDGs (2). Worldwide, the coverage of SBAs has increased by 34% since the year 2000, and currently about 83% of births are attended by skilled health personnel (12). Global MMR has dropped over the same period (1, 4, 13). However, reductions in MMR were incommensurate to increases in SBA, a mismatch that raised concerns about whether skilled personnel are measured reasonably in the same way between countries (4, 14–16).

Sudan has invested in the training of various health providers (doctors, nurse-midwives, health visitors, and midwives) to scale up access to essential maternal health services and to replace traditional birth attendants (17). All trained health providers were assumed to be SBAs and, thus, contributed towards national figures of SBA (18–20). However, locally certified midwives included in the Sudan Household Surveys, differ from other trained health providers as they are not educated, trained and regulated to international standards (21, 22). Indeed, they were not recognised internationally as midwives, instead they were classified as auxiliary midwives in the state of the world's midwifery report (21). No national assessments that assessed whether they meet criteria of SBA were available. The training of these locally certified midwives was short (∼1 year), their curriculum was not standardised, they received limited practical training, and studies have identified that a majority were functionally illiterate (23, 24). Thus, there is uncertainty about their level of competence and whether they should be classified with SBA in national records.

An accurate understanding of the workforce attending births in Sudan is currently lacking. Such an understanding provides valuable insights into the maternal health care needs that can be used to improve health and accelerate progress towards national and global targets. This study examines trends in birth attendants, using a robust categorisation of providers, by analysing the latest available, national-level data (2006–2014). It also investigates the correlation between birth attendants with maternal mortality ratio at the state level in 2016.

## 2. Methods

We used two data sources for our analyses: the Sudan household surveys (SHHS) of 2006, 2010, and 2014, and the maternal death surveillance and response report of 2016. The household surveys are nationally-representative, cross-sectional surveys, and modified versions of the widely known multiple indicator cluster survey (18). The surveys collected data on health indicators through their 2006, 2010, and 2014 iterations. However, the 2018 survey did not materialise, and no new surveys have taken place for nearly a decade (25). An upcoming survey that is currently in the survey design stage is planned, but it remains uncertain when this will take place given the protracted political, economic, and social crisis the country is undergoing (25–28). All surveys used the same two-stage, stratified, and clustered sampling method. The anonymised datasets for the 2010 and 2014 surveys are freely accessible online through the UNICEF website: http://mics.unicef.org/surveys. The 2006 survey dataset is not available online and was obtained directly from the Federal Ministry of Health in Sudan.

In our study, the main outcome was birth attendants. Women were asked to name the type of provider who attended their last childbirth from a comprehensive list if they were married or previously married, and if the birth was in the two years prior to the interview. Based on women's responses we classified birth attendants into (1) skilled birth attendants, which included doctors, health visitors, and nurse midwives, (2) locally certified midwives and (3) traditional birth attendants (TBA). This classification served two purposes: first, to capture differences between SBA and locally certified midwives in terms of skill level and training, and second, to reduce the risk of misclassifications. Women are likely able to distinguish locally certified midwives from TBAs as the former wore a white uniform and had a medical bag. However, the risk of misclassification between skilled midwives and locally certified midwives persists and is addressed further in the discussion section. All surveyed women for whom outcome data were available were included in our analyses. The term SBA, rather than skilled health personnel, will be used throughout our paper to maintain consistency with the term used in the SHHS reports that were released prior to the 2018 joint statement defining skilled health personnel (11).

**Figure 1:**
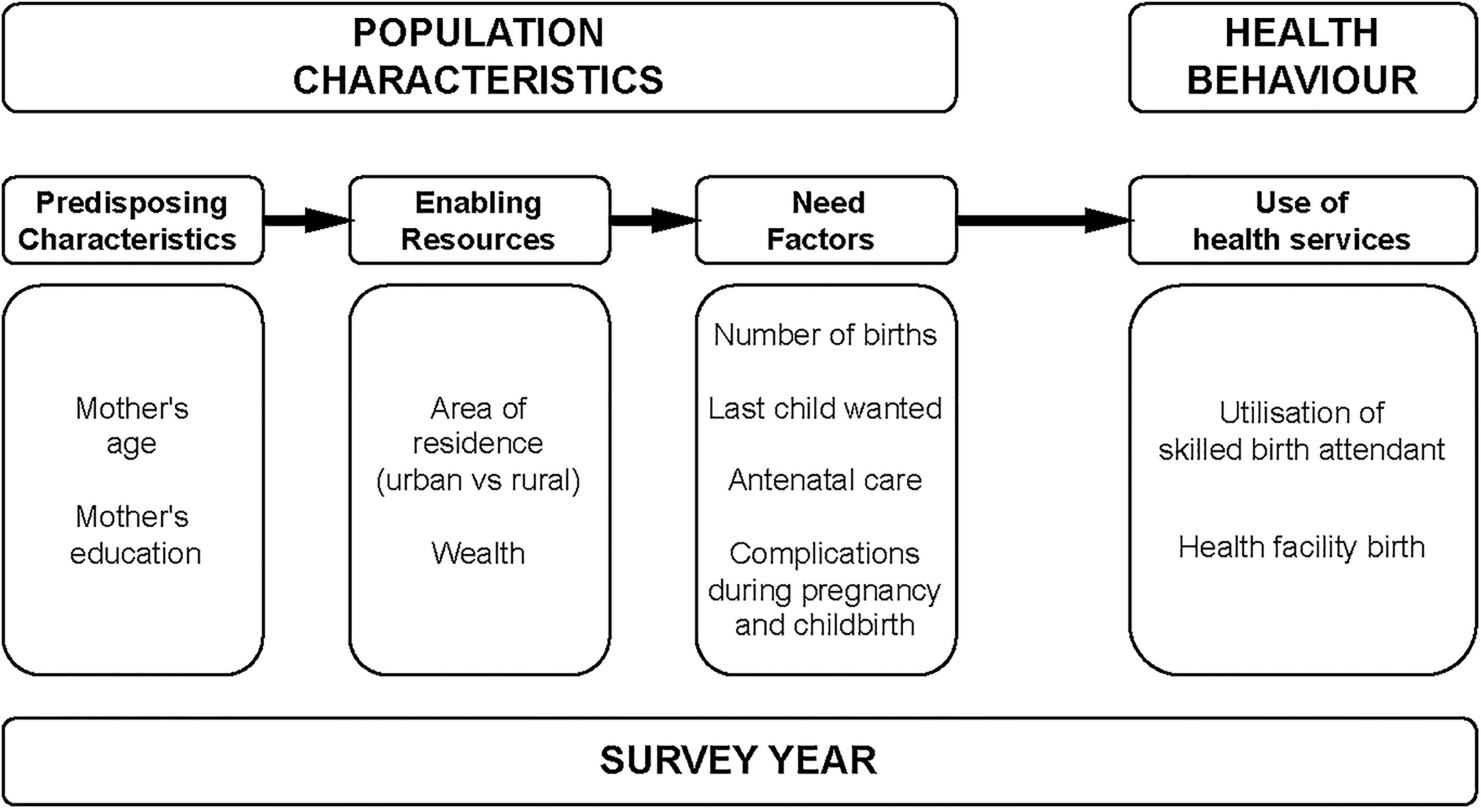
A modified Andersen-Newman framework for service utilisation in Sudan (32).

The three surveys were merged into one dataset, and a variable labelled “*surveyyear*” was generated to denote the survey year. Potential confounders that were identified from the literature and were available in the dataset were used (29–31), these were: maternal age at the time of the survey, mother's level of education, birth order of last child, place of residence (urban vs. rural), wealth index, use of antenatal care, whether the pregnancy of the last birth was wanted, and mode of delivery (vaginal vs. forceps, ventouse, or a Caesarean section). A modified version of the widely used Andersen and Newman framework of health services utilisation was used to conceptualise the relationship between survey year, confounders, and the outcome- See Figure 1 (32–34).

Summary statistics were calculated for confounders in each survey round and a chi-squared test for trends with one degree of freedom was used to assess change across the survey years. The weighted proportions and their 95% confidence intervals for the distribution of birth attendants were calculated for each survey year. Confounders that were statistically associated with the outcome at the univariable level at p≤0.05 or were theoretically important were adjusted for in the multivariable models. Multivariable logistic regression was used to investigate the general trends in births attended by SBAs, locally certified midwives, and TBAs. The outcome variable for each regression model compared one health provider to all others, for example, SBAs vs. locally certified midwives and TBAs. A further analysis was carried out to investigate the trends in birth attendants by place of birth (home vs. health facilities). The term community births will be used in this paper to refer to births taking place outside of a health facility setting, namely at women's homes. The final goodness-of-fit of the models was assessed using a modified Hosmer-Lemeshow test that is appropriate for clustered data. All analyses accounted for the stratified, clustered nature of the data.

The second analysis examined the correlation between SBAs, locally certified midwives, TBAs by place of birth and maternal mortality ratio (MMR) at the state level. Data on maternal deaths were collected through the maternal death and surveillance programme, and MMRs were obtained from the publicly available Maternal Death Surveillance and Response report for the year 2016 (35). The outcome variable, MMR, did not follow a normal distribution, so we used Spearman's rank correlation coefficient. All statistical analyses were conducted using Stata version 16, SE (StataCorp LLC, College Station, Texas).

## 3. Results

### 3.1. Distribution and change in the characteristics of women (2006–2014)

**Figure 2:**
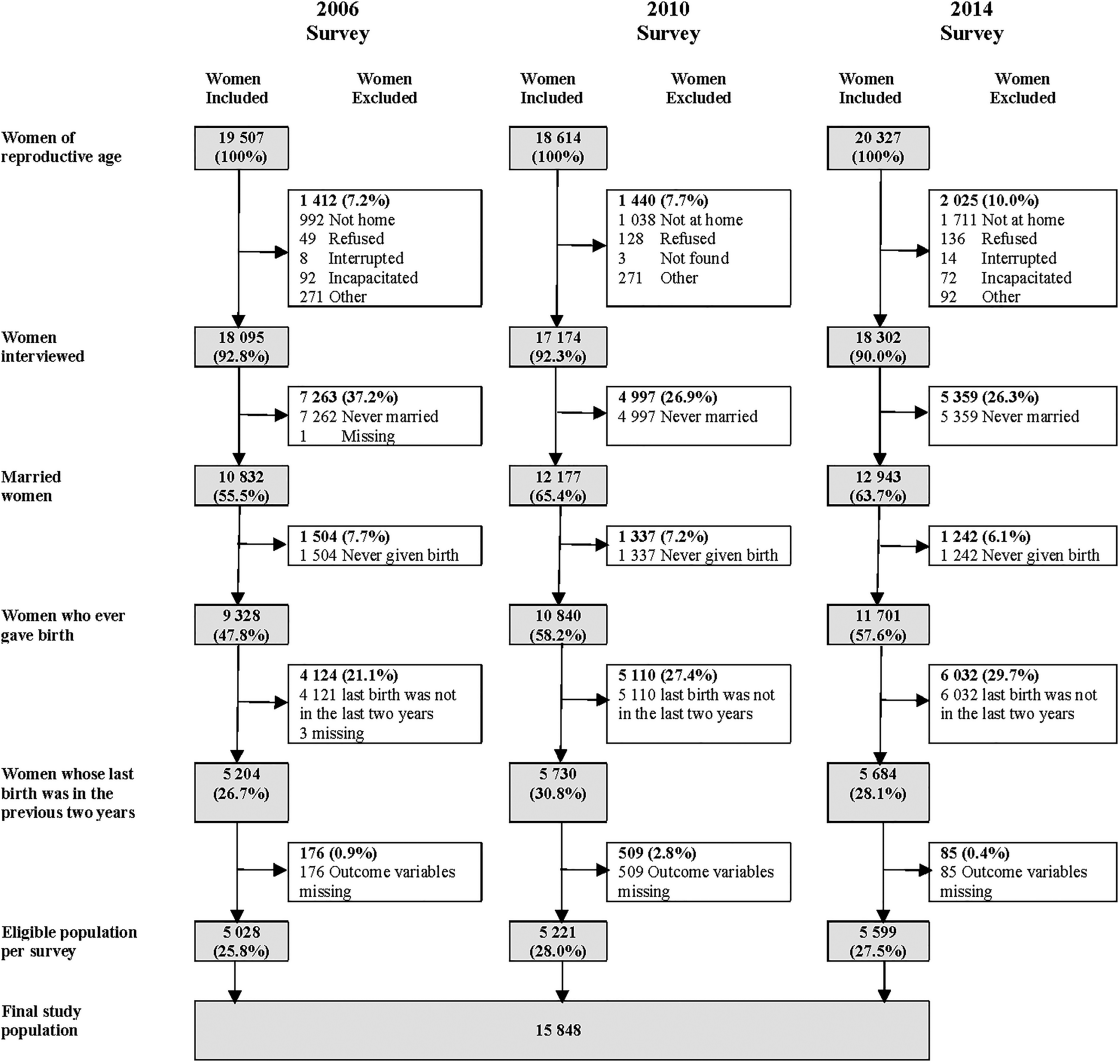
The derivation of the final study population. [Data source: Sudan Household Surveys]. Denominator for all percentages is the number of women in reproductive age for that survey year.

A total of 15,848 women were included in our analyses (Figure 2). In each survey year, the majority of women were between 25 and 29 years of age, had no formal education, lived in rural areas, had 2 to 4 children, gave birth to their last child at home, and their last childbirth was a vaginal birth. We observed statistically significant changes in the characteristics of women between 2006 and 2014: there was a modest decrease in the proportion of teenage mothers (8.3%–6.8%) and an increase in the proportions of women who had secondary education or above (18.6%–24%), lived in rural areas (64.2%–73.5%), had five or more childbirths (33.6%–40.4%), gave birth in a health facility (21.2%–28.1%), and who had an instrumental birth or a caesarean section (6.8%–9.3%). Table 1 shows the distribution of these key characteristics by survey year, as well as the changes in the characteristics.

**Table 1:** Distribution and change in the characteristics of women, per survey year, using Sudan Household Survey for 2006, 2010, and 2014.

	Survey year 2006		Survey year 2010		Survey year 2014		*P*-value
(*N*)	5,028		5,221		5,599		
Variables	*N*	[% (95%CI)]	*N*	[% (95%CI)]	*N*	[% (95%CI)]	
Mother's age, years							
15–19	470	8.3 (7.3–9.5)	373	7.5 (6.7–8.4)	393	6.8 (6.0–7.7)	<0.0001
20–24	1,137	22.5 (21.0–24.1)	1,180	23.0 (21.6–24.5)	1,133	20.0 (18.6–21.5)	
25–29	1,441	28.6 (26.9–30.4)	1,489	29.0 (27.5–30.6)	1,606	28.8 (27.2–30.3)	
30–34	1,016	21.0 (19.5–22.5)	942	17.0 (15.7–18.2)	1,184	21.2 (19.9–22.7)	
35+	964	19.6 (18.1–21.3)	1,237	23.6 (22.2–25.1)	1,283	23.2 (21.8–24.7)	
Mother's education							
None	2,650	49.8 (46.7–52.9)	2,576	47.3 (44.0–50.5)	2,476	39.9 (36.6–43.3)	0.0003
Primary	1,599	31.6 (29.3–34.0)	1,683	33.1 (30.9–35.5)	1,927	36.1 (33.5–38.7)	
Secondary	608	14.2 (12.2–16.4)	724	14.4 (12.7–16.2)	851	16.7 (15.0–18.6)	
Tertiary or above	171	4.4 (3.4–5.7)	238	5.3 (4.2–6.6)	344	7.3 (6.0–8.9)	
Missing	–	–	–	–	1	–	
Area of residence							
Rural	3,561	64.2 (60.0–68.3)	3,740	73.0 (68.7–76.8)	4,121	73.5 (69.6–77.2)	0.009
Urban	1,467	35.8 (31.7–40.1)	1,481	27.0 (23.2–31.3)	1,478	26.5 (22.9–30.4)	
Wealth index							
First (Poorest)	507	10.4 (8.7–12.2)	1,096	23.7 (20.7–27.0)	1,223	22.3 (19.1–25.9)	<0.0001
Second	1,012	19.2 (17.3–21.2)	1,208	21.8 (19.7–24.1)	1,507	21.8 (19.4–24.4)	
Third	1,451	26.5 (24.5–28.7)	1,238	21.6 (19.5–23.9)	1,272	21.2 (18.7–23.9)	
Fourth	1,288	25.2 (22.8–27.7)	1,002	19.6 (17.2–22.2)	903	19.5 (16.4–23.1)	
Fifth (Richest)	770	18.8 (16.2–21.5)	677	13.3 (11.0–15.9)	694	15.2 (12.7–18.1)	
Number of children							
1	1,050	19.9 (18.3–21.4)	946	18.5 (17.1–19.8)	906	16.1 (14.9–17.4)	<0.0001
2–4	2,299	46.6 (44.7–48.5)	2,312	44.9 (43.3–46.4)	2,426	43.5 (41.8–45.2)	
5+	1,679	33.6 (31.9–35.4)	1,963	36.7 (35.1–38.3)	2,267	40.4 (38.6–42.2)	
Wanted last child							
Yes	–	–	4,371	83.0 (81.5–84.4)	4,541	80.4 (78.5–82.1)	0.3
No	–	–	850	17.0 (15.6–18.5)	1,049	19.5 (17.8–21.4)	
Missing	–	–	–	–	9		
Mode of delivery							
Vaginal birth	4,733	93.2 (91.7–94.4)	4,854	92.7 (91.4–93.7)	5,099	90.7 (89.3–91.9)	0.02
Instrumental/ CS	295	6.8 (5.6–8.3)	358	7.2 (6.2–8.5)	498	9.3 (8.1–10.7)	
Missing	–	–	9	0.1 (0.07–0.3)	2	–	
Place of birth							
Health facility	891	21.2 (18.5–24.3)	1,169	22.4 (20.0–24.9)	1,431	28.1 (25.2–31.1)	0.002
Home	4,137	78.8 (75.7–81.5)	4,052	77.6 (75.1–80.0	4,168	71.9 (68.9–74.8)	
Any antenatal care use							
Yes	3,985	81.9 (79.8–83.7)	4,144	79.7 (77.6–81.7)	4,555	80.7 (78.1–83.1)	0.5
No	1,038	18.2 (16.3–20.2)	1,077	20.3 (18.3–22.4)	1,043	19.3 (16.9–21.9)	
Missing	5		–		1		
Antenatal complications^a^							
Yes	2,165	43.6 (41.2–45.9)	2,018	40.1 (37.7–42.6)	–	–	0.06
No	2,851	56.3 (53.9–58.6)	3,184	59.7 (57.2–62.0)	–	–	
Missing	12	0.2 (0.01–0.4)	19	0.2 (0.1–0.5)	–	–	
Labour complications^b^							
Yes	2,105	43.2 (40.8–45.5)	1,990	39.0 (36.8–41.3)	–	–	0.02
No	2,918	56.7 (54.4–59.1)	3,215	60.8 (58.5–63.0)	–	–	
Missing	5	0.08 (0.03–0.2)	16	0.2 (0.1–0.4)	–	–	

%: weighted proportions of women, Dash: the variable was not available in that survey round, *P*-value: A chi-squared statistic test for trends.

^a^ Pregnancy complication: excessive vaginal bleeding, high fever, convulsions -not from fever, and jaundice.

^b^ Labour complications: prolonged labour, excessive vaginal bleeding, high fever, and convulsions -not from fever.

### 3.2. General trends in birth attendants (2006–2014)

Overall, births attended by locally certified midwives accounted for most births and were the only group to increase in proportion from 36.3% (95%CI: 33.7%–38.9%) in 2006 to 55.5% (95%CI: 52.4%–58.6%) in 2014 (Table 2). Birth attended by SBAs and TBAs reduced by a third over that period, they respectively attended (22.8%; 95%CI: 20.0%–26.0%) and (21.7%; 95%CI: 18.7%–25.0%) of all births in 2014.

**Table 2:** Unadjusted and adjusted odds ratios for trends in birth attendants from 2006 to 2014.

	Birth attendants						Skilled birth attendant (vs. locally certified midwives and traditional birth attendants)		Locally certified midwives (vs. skilled birth attendants and certified midwives)	
	Skilled birth attendants		Locally certified midwives		Traditional birth attendants		Unadjusted odds ratio	Adjusted^a^ odds ratio	Unadjusted odds ratio	Adjusted^a^ odds ratio
Survey year	*N*	% (95%CI)	*N*	% (95%CI)	*N*	% (95%CI)	(95%CI)	(95%CI)	(95%CI)	(95%CI)
2006	1,555	32.6 (28.6–37.1)	1,866	36.3 (33.7–38.9)	1,607	31.1 (28.0–34.3)	1.0 (baseline)	1.0 (baseline)	1.0 (baseline)	1.0 (baseline)
2010	1,278	24.1 (21.1–27.4)	2,758	53.7 (50.7–56.6)	1,185	22.2 (19.1–25.8)	0.66 (0.54–0.80)	0.70 (0.58–0.85)	2.03 (1.72–2.39)	1.92 (1.61–2.30)
2014	1,145	22.8 (20.0–26.0)	3,130	55.5 (52.4–58.6)	1,324	21.7 (18.7–25.0)	0.61 (0.50–0.74)	0.46 (0.37–0.56)	2.19 (1.85–2.59)	2.19 (1.82–2.63)

Data source: Sudan Household Surveys of 2006, 2010, and 2014.

Percentages and their 95% confidence intervals are presented.

^a^ Adjusted for woman's age, education, place of residence, wealth index, birth order of the index birth, use of antenatal care services, and mode of delivery.

We found that women were around two times more likely to give birth attended by a locally certified midwife in 2010 and 2014 compared with 2006 after adjustment for confounders [adjusted odds ratio (aOR):1.92; 95%CI: 1.61–2.30] and (aOR: 2.19; 95%CI: 1.82–2.63), respectively. By contrast, they were less likely to give birth attended by a SBA (aOR: 0.70; 95%CI: 0.58–0.85) in 2010 and (aOR: 0.46; 95%CI: 0.37–0.56) in 2014- Table 2.

### 3.3. Trends in birth attendants by place of birth (2006–2014)

Births attended by locally certified midwives increased both in community and health facility settings while births attended by SBA or TBA generally decreased. Between 2006 and 2014, births attended by locally certified midwives increased from 33.2% (95%CI: 30.8%–36.0%) to 47.3% (95%CI: 44.5%–50.5%) in the community and from 3.2% (95%CI: 2.3%–4.4%) to 8% (95%CI: 6.9%–9.4%) in health facilities. By contrast, a significant drop was noted in the proportions of births attended by SBAs in the community from 15% (95%CI: 13.4%–16.9%) in 2006 to 3% (95%CI: 2.3%–3.7%) in 2014. There was no statistically significant change in the proportions of births attended by SBAs in health facilities although they accounted for 20% (95%CI: 17.6%–24.4%) of all births in 2014.

In our adjusted models comparing births in 2014 with births in 2006, we observed the following trends: Women giving birth in a health facility were 55% less likely to be attended by a SBA (aOR: 0.45; 95%CI: 0.31–0.65); women giving birth in the community were 83% less likely to be attended by a skilled provider (aOR: 0.17; 95%CI: 0.12–0.23) but over three times more likely to be attended by a locally certified midwife (aOR: 3.42; 95%CI: 2.74–4.27)- (Table 3).

**Table 3:** Unadjusted and adjusted odds ratios for trends in birth attendants by place of birth from 2006 to 2014.

	Facility births		Home births			
	Skilled birth attendant (vs. locally certified midwives)		Skilled birth attendant (vs. locally certified midwives and traditional birth attendants)		Locally certified midwives (vs. skilled birth attendants and traditional birth attendants)	
	Unadjusted Odds Ratio	Adjusted^a^ Odds Ratio	Unadjusted Odds Ratio	Adjusted^a^ Odds Ratio	Unadjusted Odds Ratio	Adjusted^a^ Odds Ratio
Survey year	(95%CI)	(95%CI)	(95%CI)	(95%CI)	(95%CI)	(95%CI)
2006	1.0 (baseline)	1.0 (baseline)	1.0 (baseline)	1.0 (baseline)	1.0 (baseline)	1.0 (baseline)
2010	0.91 (0.62–1.34)	1.01 (0.67–1.52)	0.35 (0.28–0.44)	0.35 (0.28–0.44)	2.43 (0.62–1.3)	3.32 (2.66–4.14)
2014	0.50 (0.35–0.72)	0.45 (0.31–0.65)	0.18 (0.14–0.24)	0.17 (0.12–0.23)	2.66 (2.14–3.30)	3.42 (2.74–4.27)

Data source: Sudan Household Surveys of 2006, 2010, and 2014.

Percentages and their 95% confidence intervals are presented.

^a^ Adjusted for woman's age, education, place of residence, wealth index, birth order of the index birth, use of antenatal care services, and mode of delivery.

### 3.4. Correlation of birth attendants by place of birth with maternal mortality ratio at the state level

The Spearman rank correlation coefficients were calculated for the relationship between the different birth attendants by place of birth and MMR, as shown in Table 4. For facility births attended by SBA and MMR there was a significant negative correlation (rho = −0.55, p: 0.02), indicating that as the proportion of births attended by SBA in a health facility increased at the state level, MMR decreased. Although the correlation between facility births attended by locally qualified midwives and MMR generally followed the same direction, it was not statistically significant. The correlation between home births attended by SBA and home births attended by locally qualified midwives and MMR showed a positive correlation, but both were not statistically significant. Lastly, a statistically significant positive correlation (rho = 0.53, p: 0.02) was observed between births attended by TBA at home and MMR.

**Table 4:** Correlation between birth attendants by place of birth with maternal mortality ratio at state level in Sudan 2016.

Variable at state level	Correlation with MMR (rho)	*P*–value
Skilled birth attendance in health facilities	−0.55	0.02
Locally certified midwives in health facilities	−0.34	0.16
Skilled birth attendance at home	0.32	0.20
Locally certified midwives at home	0.24	0.35
Traditional birth attendants	0.53	0.02

Data source: Sudan Household Surveys of 2014 and the maternal death and surveillance response for the year 2016.

## 4. Discussion

This study investigated the changes in birth attendants in Sudan (2006–2014) and the relationship between birth attendants based on the place of birth and the maternal mortality ratio at the state level. We identified changes in the maternity population over that period. Most births were attended by locally certified midwives, which increased over the analysed period both in facility and out-of-facility settings. Births attended by SBAs and TBAs had decreased over the same period. Births attended by SBAs at health facilities were associated with a lower maternal mortality ratio at the state level.

We identified a shift towards a more educated maternity population over the analysis period. The proportion of women who have completed secondary education and beyond increased by 30% (from 18.6% to 24%). Such a change is promising because individuals' utilisation of health services is influenced by their sociodemographic characteristics (32). Women with higher educational attainments are more aware of the importance of seeking healthcare during pregnancy and childbirth. Moreover, they are better empowered to navigate the healthcare system compared to women with no or low educational attainments (36–38). Indeed, studies have shown that Sudanese women who have attained secondary education or above are more likely to utilise family planning or antenatal care services (39, 40). Additionally, educated women are less likely to have teenage pregnancies (41, 42). This could potentially explain the reduction in the proportion of mothers aged 15–19 years old observed here (8.3% to 6.8%). Given that only a quarter of the maternity population in our study had achieved secondary education or above, a huge opportunity to invest in future improvements in maternal health would be lost if measures are not put in place to keep Sudanese girls in school until they complete their secondary education.

An important finding of our study is that the utilisation of SBAs (doctors, nurse midwives, and health visitors) in the community has decreased dramatically from 2006 to 2014 (aOR: 0.17; 95%CI: 0.12–0.23). This finding is potentially explained by two main reasons: firstly, a shrinking health visitor cadre. Health visitors are the main skilled providers attending births in the community. However, a lack of consistent funding has resulted in recurrent interruptions to their training since the establishment of the Institute of Health Visitors in 1948 (23, 43). As a result, few qualified health visitors have joined the health workforce over the years while many have left for reasons such as retirement (43–45). Secondly, a shift in the responsibilities of health visitors from clinical practice towards teaching and administrative roles was noted. Health visitors have been assigned supervisory duties over locally certified midwives, and their roles further included the training of locally certified midwives and the management of midwifery schools (22, 23, 46). Practicing health visitors are often stretched, and many struggle to fulfil their supervisory duties that often cover geographically widespread regions (46, 47). This likely left health visitors with limited time and resources to attend births in the community.

In this study, we have identified locally certified midwives as a significant maternal health cadre in Sudan. In 2014, they attended more than half of all births and were three times more likely to attend births in the community than in 2006 (aOR: 3.42; 95%CI: 2.74–4.27), effectively replacing other birth attendants. The training of this important cadre has historically attracted older applicants (40+ years old) with basic, if any, educational background and many were previously traditional birth attendants (23, 43, 47). The low literacy levels of students limited the scope of training they could receive (47). Studies assessing the care provided by certified midwives found that they have retained knowledge and skills directly relevant to labour care (23, 48), but had limited knowledge and skills in aspects they did not encounter regularly, such as post-abortion care (24). Several training-based interventions to upskill certified midwives in Sudan were implemented, and they reported good outcomes following the initial implementation (49–51). However, data on the long-term outcomes or impact on maternal survival of these one-off trainings are not available. In other low- and middle-income countries, providers not considered to be SBAs were able to provide good quality care when appropriate training and support were provided (52–55). Together, this suggests that for certified midwives to provide safe care for Sudanese women in the community, continuous training, supervision by SBAs, and linkages to health facilities for referring women who cannot be managed in the community are necessary. Fostering such a supportive environment has been fraught with challenges in Sudan (46), but without such an environment within which certified midwives practice, a major cadre would be underutilised and the care they provide to women would be compromised.

No national strategies were identified to upskill certified midwives to take up responsibilities in health facilities in Sudan. Yet, births attended by certified midwives in health facilities accounted for 8% of all births in 2014, a 150% increase from 2006. This suggests that the chronic and critical shortages in skilled providers in Sudan have prompted local decision-makers to employ locally certified midwives to support SBA with service delivery in health facilities. If certified midwives in health facilities work with support and supervision from SBAs, this can result in good quality maternal care (56). But if the shortages are so severe that no SBAs are available, then certified midwives could take up roles beyond their level of competence. This can have serious implications for women seeking health facility care in regions where most women give birth at home because they are likely to only seek care as a result of a complication that could not be managed at home (57, 58).

Since the last survey round of the SHHS in 2014, two new training programmes for midwives were introduced in Sudan to advance midwifery practice: (1) community midwives who complete a 2-year apprenticeship programme, and (2) professional midwives who complete a 4-year university Bachelor of Science in Midwifery (47). A recent study that assessed the competencies of community midwives identified low knowledge in diagnosing high-risk pregnancies requiring referral for health facility births and in measuring women's vital signs (47). It is likely that they do not meet international definitions of midwives and of SBAs. Limited data are available regarding the competencies and performance of professional midwives working in health facilities. However, these are recognised nationally and internationally as SBAs and midwives (21). The contribution of these cadres to the midwifery workforce in Sudan remains unclear, and further research that includes them is needed to continue monitoring trends in SBAs in Sudan.

Our ecological analyses revealed two significant relationships: an inverse relationship between facility births attended by SBAs and MMR, and a direct relationship between home births attended by TBAs and MMR. This indicates that in Sudan both the type of provider and the place of birth play an important role in improving maternal outcomes. Further research that uses individual-level data is needed to clarify the findings from our ecological analyses, especially on the relationship between locally certified midwives working in different settings and maternal outcomes.

Since the first submission of this manuscript, the protracted political unrest in Sudan has escalated. The recent and ongoing conflict has severely disrupted the health care system, rendering a substantial proportion of health facilities in the affected regions inoperative due to structural damage, lack of essential supplies, displacement of staff, or safety and security concerns (6, 59). For the maternity population, this has magnified the inaccessibility of maternal health services such as access to preventative services, antenatal care, SBAs, and facility births. Midwives continue to assist births at home despite significant challenges and risks to their safety and without support from facilities in cases of complications (60, 61). Current events will undoubtedly increase pregnant women's risks of maternal mortality and morbidity as reported in other humanitarian settings (62).

Immediate action is needed to protect pregnant women and facilitate their access to healthcare. Similarly, immediate action is necessary to safeguard healthcare providers who deliver life-saving medical interventions. In the long-term, strategies to increase the numbers and coverage of SBAs, improve access to health facilities, and evaluate the care provided by locally certified midwives both in the community and health facilities are needed. The uncertainty surrounding the skills and capabilities of locally certified midwives presents challenges to improving their competencies and subsequently the services they provide. Investments are also needed to support certified midwives working in the community and strengthen their links to SBAs and to health facilities.

A major strength of the study is the detailed categorisation of birth attendants in Sudan that improves our understanding of the diminishing contribution of SBAs. Without such categorisation, our analyses would have tremendously overestimated the role of SBAs. Indeed, locally certified midwives should be grouped separately from SBAs in government records and in epidemiological research to accurately depict care provision. Future SHHS reports should exclude certified midwives when presenting data for SBAs. Another strength of our study is that we leveraged the comparability of the three consecutive and nationally representative surveys to investigate trends. Lastly, the findings of our study are generalisable and reflect national practice during the observed period.

Our study had some limitations that need to be considered when interpreting our findings. Data were based on women's self-report of birth attendants, it required women to know the qualifications of their provider, which introduces a risk of misclassification. This is particularly an issue in facility births where more than one provider might be involved. There was a risk of recall bias as women had to remember the birth attendant for up to two years. Also, the survey used in our study mostly captured the experience of married women, thereby introducing selection bias and offering little insight into the experience of unmarried women. The last round of SHHS was in 2014, thus there is a need to use recent data that is reflective of contemporaneous trends.

## 5. Conclusions

The reducing contribution of SBAs threatens to undo decades of investments into reducing maternal deaths in Sudan. Thus, coordinated national action is needed to train and retain SBAs and to improve access to health facilities to ensure women's access to quality care around childbirth. Additionally, a long-term pragmatic strategy is needed to mitigate the impacts of critical shortages in skilled providers such as through the ongoing training and supported supervision of locally certified midwives.

## Data Availability

Publicly available datasets were analysed in this study. This data can be found here: http://mics.unicef.org/surveys. The dataset from 2006 can be obtained by contacting the Sudan Ministry of Health.
